# Analysis of the *XRCC1 *gene as a modifier of the cerebral response in ischemic stroke

**DOI:** 10.1186/1471-2350-7-78

**Published:** 2006-11-06

**Authors:** Ana VC Dutra, Hsiu-Fen Lin, Suh-Hang H Juo, Harvey Mohrenweiser, Souvik Sen, Raji P Grewal

**Affiliations:** 1 New Jersey Neuroscience Institute, 65 James Street, Edison, New Jersey, 08818, USA; 2 Department of Neurology, Kaohsiung Municipal Hsiao-Kang Hospital, Kaohsiung Medical University, Kaohsiung, Taiwan; 3 Department of Neurology, Kaohsiung Medical University Hospital, Kaohsiung, Taiwan; 4 Graduate Institute of Medical Genetics, Kaohsiung Medical University, Kaohsiung, Taiwan; 5 Epidemiology Division, Department of Medicine, University of California, Irvine, California, 92697, USA; 6 Department of Neurology, University of North Carolina, North Carolina, 27599, USA

## Abstract

**Background:**

Although there have been studies of the genetic risk factors in the development of stroke, there have been few investigations of role of genes in the cerebral response to ischemia. The brain responds to ischemia in a series of reactions that ultimately influence the volume of a stroke that, in general, correlates with disability. We hypothesize that polymorphisms in genes encoding proteins involved in these reactions could act as modifiers of this response and impact stroke volume. One of the pathways participating in the cerebral ischemic response involves reactive oxygen species which can cause oxidative damage to nucleic acids. DNA repair mechanisms are in place to protect against such damage and imply a role for DNA repair genes in the response of the brain to ischemia and are potential candidate genes for further investigation.

**Methods:**

We studied two common polymorphisms in the DNA repair gene, XRCC1, C26304T and G28152A, in 134 well characterized patients with non lacunar ischemic strokes. We also performed a case control association study with 113 control patients to assess whether these variants represent risk factors in the development of ischemic stroke.

**Results:**

Independent of etiology, the "T" allele of the C26304T polymorphism is significantly associated with larger stroke volumes (T-test analysis, p < 0.044; multivariate regression analysis, β = 0.23, p < 0.008). In the case control association study, we found that neither of these polymorphisms represented a risk factor for the development of stroke.

**Conclusion:**

Our study suggests a major gene effect of the "T" allele of the C26304T polymorphism modulating the cerebral response to ischemia in non lacunar ischemic stroke.

## Background

Stroke is the leading cause of disability in North America and with an annual estimate of 4 million survivors, there are considerable emotional and financial costs in the care of patients who have suffered cerebrovascular disease [1,2]. As the population ages, the stroke rate more than doubles for each successive decade after age 55 and it is likely that morbidity will also continue to increase [3].

While there are recent research efforts exploring the role of genetic factors that could contribute to the development of stroke, there are few studies of the genetics of the cerebral response to ischemia. Studies of induced strokes in transgenic animals indicate that manipulation of certain genes can influence the resultant volume [4,5]. We hypothesize that there is variability in the cerebral response to ischemia which may be mediated by polymorphisms in genes encoding proteins that participate in this response. Polymorphisms in these genes exerting a major affect could enhance or diminish endogenous neuroprotective mechanisms and ultimately impact the volume of an ischemic stroke. This has clinical significance because, in general, the volume of a stroke correlates with the clinical severity and the resultant degree of disability of the patient [6,7].

It is recognized that cerebral ischemic injury results in a core of necrotic tissue surrounded by a penumbra of potentially salvable tissue in which neurons are functionally inactive but can be rescued [8]. The development of this ischemic penumbra is limited in time by a cascade of biochemical events which are a consequence of the initial ischemia followed by subsequent reperfusion. The reperfusion which occurs shortly after ischemia has been shown to reduce infarct volume, but at a later period may exacerbate ischemic injury [9]. It has been suggested that reperfusion increases reactive oxygen species (ROS) production which can have further deleterious consequences [10-12]. Cell exposure to these ROS, which include nitric oxide, superoxide ions and hydroxyl radicals, can result in oxidative DNA damage. DNA repair enzymes function to monitor and repair such damage [12-14].

A number of DNA repair genes have been described and broadly can be divided into base excision repair (BER), nucleotide excision repair (NER), and mismatch repair (MMR). The BER pathway involves a coordinated action of many core genes [15]. The X-ray repair cross-complementing group 1 (XRCC1) gene-product plays a central role in the DNA BER pathway by interacting with DNA ligase III, DNA polymerase beta and poly (ADP-ribose) polymerase [16]. The XRCC1 protein rejoins DNA strand breaks and repairs gaps left during BER [17]. XRCC1 gene expression levels are high in the brain and there is evidence of involvement of this gene in cerebral tissue injury [18].

We hypothesize that biologically significant polymorphisms in the XRCC1 gene could result in variability in DNA repair capacity and modify the response of the brain to cerebral ischemia ultimately influencing infarct volume. We selected polymorphisms in this gene for study based upon conservation of the gene sequence through evolution, frequency, and those occurring in exons resulting in amino acid changes of potential functional significance [19]. Two common polymorphisms, the C26304T (exon 6, Arg194Trp) and G28152A (exon 10, Arg399Gln) in the XRCC1 gene were studied both as risk factors for the development of stroke and as modifiers of the response of the brain to ischemia.

## Methods

### Subjects

We characterized the clinical and demographic data on 134 patients admitted with non-lacunar ischemic infarcts to the stroke unit at John F. Kennedy Medical Center, Edison, New Jersey. In addition, 113 healthy age, sex and ethnically matched controls were recruited from local physician offices. Strokes were categorized according to the Trial of ORG 10172 in Acute Stroke Treatment (TOAST) [20] and Oxfordshire criteria [21]. In the majority of patients, (79%) diffusion MRI scans were available for review, alternatively, cerebral CT scans (21%) were used to measure stroke lesion volume. Patients given TPA were excluded from the study. Infarction volume was estimated by averaging the calculated values obtained from direct measurements of the cerebral images by two examiners by previously described methods [22]. All study protocols and methods were approved by the local Institutional Review Board (IRB) at JFK Hospital.

### Samples for variation screening

The samples screened for variation were the first 92 samples from the "DNA Polymorphism Discovery Resource" at the Coriell Institute for Medical Research (Group I). This resource was developed by the NIH in order to have a common set of samples available to investigators screening for common variants existing in the general population of the United States [25]. The samples are from U. S. residents selected to represent the major ethnic groupings of the population, although the ethnic origin of specific individuals is unknown. The individuals in this sample set are from population groups as follows: European-American 23; African-American 23; Mexican-American 11; Native-American 11; Asian-American 23. As all samples came from commercially available cell lines from healthy anonymous donors, the protocol was considered exempt by the Lawrence Livermore National Laboratory Review Board under 10CRF745.101 (b).

### Identification of XRCC1 variants

The resequencing strategy and data analysis methods employed to identify variants is described elsewhere in detail [23,24]. Briefly, this involved the sequencing of the same genomic region in the 92 individuals in order to identify DNA sequence variation. PCR products containing the individual exons of XRCC1, plus adjacent intronic and non-coding regions were generated using oligonucleotide primers specific to XRCC1. It should be noted that complete intronic and untranslated regions were not sequenced in this study.

### Variants genotyped

Two common amino acid substitution variants in XRCC1 were genotyped in this study. The Arg to Trp substitution at residue 194 and nucleotide 685 (C/T) of cDNA reference sequence NM006297 is located at nucleotide 48749414 of the UCSC Genome Browser Golden Path assembly of May 2004 and is SNP ID rs1799872 of dbSNP. The Arg to Gln substitution at residue 399 is nucleotide 1301 (G/A) located at nucleotide 48747566 of the May 2004 assembly of Golden path and SNP ID rs25487 in dbSNP.

### Genotyping

Genomic DNA was isolated from blood samples (Puregene Systems, Gentra). Standard PCR protocols, followed by restriction enzyme digestions were used to genotype the XRCC1 polymorphisms, as previously described [26]. Briefly, the primer pairs used were: C26304T, F 5'AGCTGTACCTGTCACTCCCC 3'and R 5' CCCTACTCACTCAGGACCCA 3'; G28152A, F GCCCCTCAGATCACACCTAA 5' and R 5'ATTGCCCAGCACAGGATAAG 3'. The PCR conditions were modified from those published and consisted of an initial melting temperature of 94°C for 4 minutes followed by 30 cycles of melting (94°C, 30 s), annealing (64°C, 45 s) and extension (72°C, 45 s). A final extension step (72°C, 7 min) terminates the reaction (Perkin-Elmer 9700). Ten ul of the PCR products were subjected to restriction enzyme digestion for 15 hours according to standard conditions for each enzyme provided by the supplier (New England Biolabs, MA). The C26304T polymorphism was analyzed after digestion with Pvu II and the G28152A variant with Msp I. Ten μl of the digested products were resolved by gel electrophoresis (6% polyacrylamide gel). The genotypes obtained by this method were confirmed by direct sequencing. The allele frequency was obtained by direct gene counting.

### Statistical analysis

Observed numbers of each genotype was compared with those expected under Hardy-Weinberg equilibrium (HWE) by using the x^2 ^test.

To investigate genetic susceptibility to stroke, we used the 2 × 3 contingency table for the x^2 ^test or Fisher exact test to determine whether genotype distributions were different between cases and controls. In addition, we also tested for any significant difference of allele frequencies between cases and controls in a 2 × 2 table. To study the correlation between these two polymorphisms and stroke volume, ANOVA was used to test for differences between genotypes and mean stroke volume. Multivariate regression was carried out to study other covariates that may be related to stroke volume. We evaluated the strength of linkage disequilibrium (LD) between the two polymorphisms by Haploview [27]. The PHASE program was employed to infer haplotypes and test for the association between phenotypes and haplotypes/diplotypes [28].

## Results

Among the patients, applying the TOAST classification, the etiology of the non-lacunar ischemic strokes included 61 large artery atherothrombosis, 40 cardioembolic and 33 with other known or unknown causes (Table 1). The infarct volume averaged 75 cm^3 ^and ranged between 0.2 cm^3 ^to 482 cm^3^.

**Table 1:** Demographic and clinical characteristics of the patients

Female n (% female)	73 (54.5)
Mean age, years (SD ^a^)	70.8 (12.9)
Ethnicity n (%)	
Caucasians	93 (69.9%)
African Americans	12 (9.0%)
Others ^b^	29 (21.6%)
Clinical characteristics ^c^	
Hypertension	100 (74.6%)
Diabetes	54 (39.1%)
Atrial Fibrillation	36 (27.3%)
TOAST Classification ^d^	
Cardioembolism	40 (29.9%)
Large-artery atherosclerosis	61 (45.5%)
Others ^e^	33 (24.6%)

^a ^S.D. – Standard deviation.

^b ^Includes Hispanic, Asian, and other ethnic groups.

^c ^Other clinical data recorded includes history of smoking, alcohol use, coronary artery disease, peripheral vascular disease, carotid stenosis, cholesterol profile, and homocysteine levels.

^d ^TOAST – Trial of Org 10172 in Acute Stroke Treatment.

^e ^Includes stroke of other determined etiology, and stroke of undetermined etiology.

The uncommon "T" allele frequency of the C26304T polymorphism was present in 8.0% of cases and 7.8% of the controls (data not shown). The less common "A" allele frequency of the G28152A polymorphism was found in 36.2% cases and 31.9% of the controls. The genotypes of the two polymorphisms did not deviate from Hardy-Weinberg equilibrium in either cases or controls. The D' of two polymorphisms was 0.7, suggesting they are in LD. Our analysis showed no differences in the frequencies or genotypes of either of the two polymorphisms between the stroke and control patients (p > 0.5).

There was only one subject with the TT genotype of the C26304T variant and so we combined the CT and TT as one group in the following analyses (Table 2). If only the CT genotype was analyzed, the results were almost identical. T- test analysis of the C26304T polymorphism showed that the (CT + TT) group was associated with a significantly larger ischemic lesion volume compared to the CC genotype (p < 0.044). Analysis of the G28152A polymorphism showed significant differences of stroke volume among the three genotypes (p = 0.02). However, including age, gender, DM, HTN, smoking and both polymorphisms in the multivariate regression model, we found only the C26304T polymorphism (regression coefficient, β = 0.23, p < 0.008) and gender (β = 0.22, p = 0.013 for males) were significantly associated with stroke volume (Table 2). Haplotype and diplotype analyses did not improve statistical significance (data not shown). Subgroup analyses focusing on the large artery atherothrombosis patients showed that the CT variant of the C26304T was still significantly associated with a larger ischemic lesion volume compared to the CC genotype (p = 0.045). In the multiple regression model for large vessel thrombosis, only the CT genotype of the C26304T polymorphism (β = 0.43, p < 0.001) and gender (β = 0.31, p = 0.014 for males) were significant in this subgroup (Table 2).

**Table 2:** Genotype and stroke volume data

SNP	Genotype	N	Mean stroke Volume(cm^3^) ± SD	β^a^	P-value^a^
All patients					
C26304T	CC	114	62.4 ± 74.5		
	CT + TT^b^	20	130.6 ± 138.6	0.233	0.008
TOAST-LAA^c^					
C26304T	CC	54	61.6 ± 80.0		
	CT	7	227.6 ± 174.3	0.434	0.001
TOAST-Non-LAA					
C26304T	CC	60	63.1 ± 68.8		
	CT + TT^b^	13	78.3 ± 82.4	0.074	0.564

^a ^After adjusting for age, gender, DM, HTN, and smoking in the multivariate regression model

^b ^TT genotype N = 1

^c ^Toast Classification-Large Artery Atherosclerosis

## Discussion

The frequencies of both the C26304T and the G28152A polymorphisms in the population we studied are comparable to published data [19,29,30]. There is no significant difference in the frequencies of either of these polymorphisms between the control and stroke patients suggesting that they do not represent a major genetic risk factor for the development of stroke.

Our data show a trend towards larger volume strokes in men. There has been little study of any potential relationship between gender and acute ischemic stroke volume in humans. Although our results are of interest, further study with a larger sample size of patients with non-lacunar strokes are needed to confirm if any gender differences in stroke volume exist.

Our results indicate that the C26304T polymorphism is associated with a significant difference in stroke volume. The "T" allele is associated with higher ischemic stroke volume independent of the etiology of the stroke. There is no significant additive effect of the G28152A polymorphism genotypes or any clinical feature.

Even though the two SNPs are in linkage disequilibrium, there is no association of stroke volume with the G28152A polymorphism either independently or in haplotype analysis. This suggests that there may be a functional consequence of this specific polymorphism rather than the effect of another tightly linked gene.

Although there are many reports studying the association of both of these XRCC1 SNP in cancer, there have been relatively few studies of their functional significance [26]. The C26304T polymorphism is located within the gene region encoding that domain of the protein which interacts with poly (ADP-ribose) polymerase that is required for DNA repair [28,29].

Oxidative DNA damage can be reversed by DNA repair mechanisms, usually within the first 30 min of reperfusion [30]. In cerebral ischemia, a temporal sequence of oxidative stress-induced DNA damage starts with the beginning of reperfusion-oxidative DNA base modifications, followed by the formation of direct DNA breaks, and finally DNA fragmentation and cell death [30]. Studies of transient cerebral ischemia in animal models suggest that an early decrease of XRCC1 protein levels and failure of the DNA repair mechanisms could contribute to DNA fragmentation [11,31-33]. A biologically plausible hypothesis is that "T" allele of the C26304T polymorphism reduces the fidelity of BER and thereby results in more ROS species. This could subsequently contribute to further oxidative DNA damage and perpetuate the cycle of cerebral cellular and tissue damage.

This is one of the few reports studying the potential genetic contribution in the cerebral response to ischemia in humans. Although the study is limited by a relatively small sample size, it is the first to demonstrate a potential major gene effect of a polymorphism in any DNA repair gene upon ischemic stroke volume. Further investigations of the XRCC1 gene as a modifier of the cerebral response in ischemic stroke in a larger sample size are needed to confirm and expand on these results.

Genes encoding proteins that are participants in the cascade of events that follow ischemia are candidates for further study. In broad terms, the results of such studies may have implications for future targeting efforts for the development of more effective neuroprotective therapies.

## Conclusion

Our study suggests that independent of stroke etiology, there a major effect of the "T" allele of the C26304T polymorphism of the XRCC1 gene in the response of the brain to non lacunar ischemic stroke.

## Competing interests

The author(s) declare that they have no competing interests.

## Authors' contributions

AVD collected patient data, performed the genotyping and prepared a draft of the manuscript. HFL and SHJ performed the genetic statistical analysis and revised the manuscript. HW provided prepublication data on the presence and epidemiology of the XRCC1 polymorphisms that were studied and also revised the manuscript. SS assisted in recruiting patients for the study and assisted in study design. RPG conceived of the idea, developed the study design and was responsible for overall supervision of all aspects of this research project. All authors read and approved of the final manuscript.

## Pre-publication history

The pre-publication history for this paper can be accessed here:


